# Optimization of translation enhancing element use to increase protein expression in a vaccinia virus system

**DOI:** 10.1099/jgv.0.001624

**Published:** 2021-08-12

**Authors:** Harold B. Richard Jr., Stephanie Minder, Amandeep Sidhu, Amber N. Juba, James K. Jancovich, Bertram L. Jacobs, Brian P. Wellensiek

**Affiliations:** ^1^​ Biomedical Sciences Program, College of Graduate Studies, Midwestern University, Glendale, AZ 85308, USA; ^2^​ Arizona College of Osteopathic Medicine, Midwestern University, Glendale, AZ 85308, USA; ^3^​ Department of Biological Sciences, California State University-San Marcos, San Marcos, CA 92078, USA; ^4^​ Biodesign Center for Immunotherapy, Vaccines and Virotherapy, Arizona State University, Tempe, AZ 85287, USA; ^5^​ School of Life Sciences, Arizona State University, Tempe, AZ 85287, USA

**Keywords:** protein expression, translation enhancing element, vaccine development, vaccinia virus

## Abstract

Since the successful use of vaccinia virus (VACV) in the immunization strategies to eliminate smallpox, research has been focused on the development of recombinant VACV strains expressing proteins from various pathogens. Attempts at decreasing the side effects associated with exposure to recombinant, wild-type viral strains have led to the development of attenuated viruses. Yet while these attenuated VACV’s have improved safety profiles compared to unmodified strains, their clinical use has been hindered due to efficacy issues in stimulating a host immune response. This deficiency has largely been attributed to decreased production of the target protein for immunization. Efforts to increase protein production from attenuated VACV strains has largely centered around modulation of viral factors, while manipulation of the translation of viral mRNAs has been largely unexplored. In this study we evaluate the use of translation enhancing element hTEE-658 to increase recombinant protein production in an attenuated VACV system. Optimization of the use of this motif is also attempted by combining it with strategies that have demonstrated effectiveness in previous research. We show that extension of the 5′ leader sequence containing hTEE-658 does not improve motif function, nor does the combination with other known translation enhancing elements. However, the sole use of hTEE-658 in an attenuated VACV system is shown to increase protein expression levels beyond those of a standard viral promoter when used with a wild-type virus. Taken together these results highlight the potential for hTEE-658 to improve the effectiveness of attenuated VACV vaccine candidates and give insights into the optimal sequence context for its use in vaccine design.

## Introduction

Poxviruses are a large family of viruses known to infect a wide variety of vertebrate and invertebrate hosts [1]. Viruses of the genus *Orthopoxvirus* are of particular interest to humans and include the variola (VARV) and vaccinia (VACV) viruses [2]. VARV is the causative agent of smallpox and was responsible for widespread, devastating illness throughout history, while VACV was eventually used as the vaccination strain in the worldwide campaign to eliminate smallpox [2, 3]. Since the use of VACV as a vaccine was such a success, and VARV was no longer naturally circulating in the human population, research turned towards modifying the use of VACV to express recombinant proteins and target other pathogens. This led to the generation of several strains of attenuated VACV, most notably the MVA, LC16m8 and NYVAC strains [4, 5]. While MVA and LC16m8 were generated via serial passage through non-human hosts, NYVAC was produced through specific engineering of the viral genome. In this highly characterized example of viral engineering, 18 open reading frames (ORFs) were deleted from the wild-type (wt) Copenhagen strain of VACV [6]. The genes contained in these ORFs are involved in the pathogenicity and host range regulation of VACV, and their removal resulted in a viral strain that is unable to effectively replicate in human cells [4, 6]. Research into the immunological characteristics of NYVAC has indicated that while the virus is immunogenic, it’s effectiveness as a vaccine candidate is limited by its replication incompetency and overall protein expression. This, in turn, has led to focused research efforts on increasing protein expression from NYVAC [4, 7]. These efforts have largely focused on the modulation of various viral factors [8, 9], while regulation of the translation involving viral RNAs remains largely unexplored.

It is known that regulation of translation initiation serves a pivotal role in cellular protein production. Both alterations of the translation machinery and specific sequences within the messenger RNA (mRNA) 5′ and 3′ untranslated regions (UTRs) have been shown to effect overall translation levels [10, 11]. The presence or absence of these mechanisms in the 5′ UTR, or leader sequence, of an mRNA is of particular interest in the regulation of translation initiation. Studies have shown that the specific length of the 5′ UTR alone is enough to influence translation [12, 13]. Furthermore, the presence of specific sequences serve as interaction points for ribosomal RNA or various proteins. These interactions may have an inhibitory or activating effect on protein translation by effecting the interaction of various parts of the ribosomal initiation complex with the mRNA [11].

Research exploring sequences which can influence translation led to the discovery of a vast number of motifs spread throughout the human genome [14]. These motifs, termed translation enhancing elements (TEEs), were shown to increase protein expression up to 100-fold when compared to random genomic sequences using a VACV-based expression system [14]. Of the TEEs evaluated, the motif termed hTEE-658 displayed higher levels of activity than any other. Further characterization of this sequence revealed its function as both a promoter and a translation enhancer in a VACV expression system, while detailed mutational analysis identified the core 37 nucleotide functional motif within the full sequence [15]. Additionally, these findings were demonstrated when driving the production of numerous proteins, highlighting the potential versatility in employing hTEE-658 for targeted protein expression.

While recombinant protein levels expressed in a wt VACV system have been shown to generate a robust immune response, the use of those viral strains in vaccination strategies leads to several safety concerns [16]. By employing hTEE-658 to increase the production of recombinant protein, the potential exists to enhance the effectiveness of attenuated VACV vaccine candidates while maintaining the modifications which increase the safety of those attenuated viral strains. The current study focuses on the potential for hTEE-658 to enhance protein production when used in combination with an attenuated VACV strain. Methods to further increase protein production driven by hTEE-658 were evaluated through the combination of the motif with extended leader sequences or with other sequences that have demonstrated translation enhancing capabilities. By determining the optimal sequence combination for the use of hTEE-658, the best candidate for further exploration into the generation of recombinant, attenuated VACV-based vaccines can be identified.

## Methods

### Cell culture and viral stocks

HeLa and BSC40 cells were obtained from American Type Culture Collection (ATCC). HeLa cells were maintained in 1× MEM (Mediatech) with 5 % fetal bovine serum (FBS, Atlanta Biologicals) and 5 µg ml^−1^ gentamicin (MP Biomedicals), while BSC40 cells were maintained in 1× DMEM (Mediatech) with 10 % FBS and 5 µg ml^−1^ gentamicin. Cultures were kept at 37 °C in a humidified atmosphere containing 5 % CO_2_. The NYVAC strain of VACV was obtained from Sanofi-Pasteur.

### Sequence construction

To evaluate the effect of leader sequence length and composition on hTEE-658 function, a random sequence generator [17] was used to produce four 30-nucleotide spacer sequences. Additionally, six sequences with demonstrated translation enhancing capabilities were selected [14, 18]. Prior to inclusion in this study, all sequences were evaluated for the presence of start/stop codons, predicted formation of secondary structure and overall GC content [19, 20]. Sequences were constructed via a combination of annealing single-stranded oligonucleotides and Klenow extension, followed by PCR to add restriction endonuclease sites for *Bam*HI and *Nco*I to the 5′ and 3′ ends, respectively.

### Luciferase reporter plasmid generation

Constructed sequences were inserted into a set of previously described luciferase reporter plasmids to determine the effects on translation levels [15]. Within these plasmids, either the vaccinia virus synthetic late promoter (slp) or hTEE-658 were used to facilitate transcription. Sequences were added to the plasmids through reciprocal insertion using restriction digestion with *Bam*HI and *Nco*I. Inserted sequences were located immediately downstream of the promoter site and immediately upstream of the coding region for the firefly luciferase protein. Correct plasmid construction was verified via sequencing at the Arizona State University Genomics Core laboratory.

### Luciferase reporter assay

A transfect-infect assay was used to evaluate the effects of inserted sequences on translation levels, as described previously [15]. In brief, 15,000 HeLa cells were seeded per well into a white, flat-bottom 96-well plate 18 h prior to transfection. Following incubation, culture media was removed and replaced with 50 µl OptiMEM (Gibco). Transfection was accomplished by the addition of transfection mix, which contained 100 ng plasmid DNA, 0.3 µl TransIT 2020 transfection reagent (Mirus BioLab) and OptiMEM to a final volume of 10 µl, with three technical replicates per sample. Cells were then immediately infected with either the wild-type Copenhagen or NYVAC strains of vaccinia virus at a multiplicity of infection (m.o.i.) of 3 PFU cell^−1^. In this assay, the vaccinia virus RNA polymerase interacts with the reporter plasmids and facilitates transcription within the cytoplasm. Cellular ribosomes then translate the mRNA generated. Cells were lysed within the plate 6 h post-infection using 1× Reporter Lysis Buffer (Promega), and luciferase activity determined via the Luciferase Assay System and a Glomax microplate luminometer (Promega). For experiments involving the use of recombinant NYVAC, 15,000 BSC40 cells were plated in a white 96-well plate 18 h prior to infection. Following incubation, cell culture media was replaced with 50 µl OptiMEM. Cells were then infected at an m.o.i. of 5 PFU cell^−1^, with duplicate technical replicates per sample. Six hours post-infection, cells were lysed within the plate and a luciferase assay performed as described above.

### RNA characterization

Total RNA was isolated from cells 6 h post-infection using the RNeasy Micro Kit (Qiagen) following the manufacturer’s instruction with the on-column DNaseI digestion. Isolated RNA was assessed for quantity and quality using a NanoDrop ND-1000 (Marshall Scientific), with the criteria that only those samples containing a A_260_/A_280_ ratio between 1.8 and 2.1 were suitable for further use. Complementary DNA (cDNA) was generated using 400 ng of isolated RNA and an oligo (dT_22_) primer with Superscript II reverse transcriptase (Fisher Scientific) at 42 °C for 1.5 h. Quantitative real-time PCR (qPCR) was used to determine mRNA levels and was conducted using the iQ^TM^ SYBR Green Supermix (Bio-Rad) following the manufacturer’s protocol with 42 ng of cDNA as template. Previously validated primer pairs were used at a final concentration of 0.25 µM each to amplify a portion of the luciferase and hypoxanthine-guanine phosphoribosyltransferase (HPRT, Entrez gene ID: 3251) coding regions [18]. Reactions were assembled in MicroAmp Fast optical 96 well plates (Applied Biosystems), which were then adhesively sealed using optical sealing tape (Bio-Rad). qPCR was conducted in a StepOnePlus real-time PCR system (Applied Biosystems), with cycling conditions as follows: 95 °C for 10 min followed by 40 cycles of 95 °C for 15 s and 60 °C for 1 min. A pre-programmed melting curve was completed following cycling and confirmed uniform amplicon formation for each primer pair. Reported luciferase mRNA levels were normalized to HPRT mRNA levels using the ΔΔCt method.

### Western blot analysis

Plasmids expressing the HIV-1 gp120 protein were constructed by replacing the luciferase gene with the gp120 gene using *Nco*I and *Spe*I restriction sites within the reporter plasmids. HeLa cells were transfected with the gp120 containing plasmids and subsequently infected with the NYVAC strain of vaccinia virus, following the transfect-infect protocol described above. Following incubation, cells were lysed with Pierce RIPA Buffer (Thermo Scientific) supplemented with Protease Inhibitors (Roche) and collected into a 1.5 ml microcentrifuge tube on ice. Thirty microliters of each sample was combined with 10 µl of 4× Laemmli Sample buffer supplemented with 10 % beta-mercaptoethanol. Proteins were denatured by heating for 5 min at 95 °C before being run on a Criterion 4–12% Bis-Tris gel (Bio Rad) for 60 min at 150 Volts. Proteins were then transferred to a nitrocellulose membrane using a Criterion Blotter (Bio Rad) at 100 Volts for 60 min. After transfer, the membrane was treated with Revert Stain Solution (LI-COR) following the manufacturers’ protocol for total protein normalization. The membrane was then blocked with gentle agitation for at least 1 h at room temperature in PBS (137 mM NaCl, 2.7 mM KCL, 8 mM Na_3_PO_4_, 20 mM K_3_PO_4_, pH 7.4) supplemented with 5 % non-fat dry milk (NFDM). Following blocking, the membrane was incubated overnight with gentle agitation at 4 °C with an anti-HIV-1 gp120 polyclonal antibody (abcam 21179) at a 1 : 1000 dilution in PBST (PBS + 0.05 % Tween 20, pH 7.4) supplemented with 5 % NFDM. After incubation, the membrane was washed vigorously three times with PBST for 5 min. Following the wash, the membrane was incubated with gentle agitation at room temperature with a secondary IRDye 800 CW Donkey-anti-goat antibody (LI-COR Biosciences 925–32214) at a 1 : 25 000 dilution in PBST supplemented with 5 % NFDM for at least 1 h at room temperature with gentle agitation. The membrane was then washed three times with PBST as described above, followed by an additional PBS only wash. The fluorescent signals were visualized using an Odyssey Clx Scanner (LI-COR Biosciences). Reported fluorescence intensity of the bands corresponding to gp120 was normalized to the fluorescence of total protein as determined by Revert staining. For experiments analyzing VACV replication in HeLa cells, cells were plated and infected with the indicated strains of VACV as above. Following cell lysis, early VACV protein expression was detected using an antibody against the viral E3 protein (generated by the Jacobs laboratory), while an antibody (abcam 35219) against total VACV proteins, which recognizes primarily late proteins, was used to detect late protein expression. All other blotting and imaging protocols were followed as above, with the exception that the antibodies were used at a 1:1000 dilution.

### Recombinant VACV construction

Expression constructs were inserted into the thymidine kinase (TK) locus of a modified, replication competent NYVAC strain [7] by homologous recombination as described previously [21]. In brief, the firefly luciferase gene was amplified from previously generated reporter plasmids via PCR, with or without the hTEE-658 sequence immediately upstream of the coding region. The 5′ and 3′ ends of the PCR products also contained a 30-nucleotide region of homology to the ‘TK right arm’ and ‘TK left arm’, respectively. These regions of homology corresponded to the TK locus within the genome of the virus, which was previously designed to contain the coding region for GFP [7]. Homologous recombination to reciprocally insert the PCR product in place of the GFP coding region within the viral genome occurred via infection of BSC40 cells, followed by transfection of the PCR products. Recombinant progeny virions were harvested 48 h post-transfection-infection and were screened for the absence of GFP expression via serial passaging in BSC40 cells. Correct insertion of the desired information was further confirmed by sequencing of the TK locus at the Arizona State University Genomics Core laboratory.

## Results

### Comparison with VACV strains

**Fig. 1.**
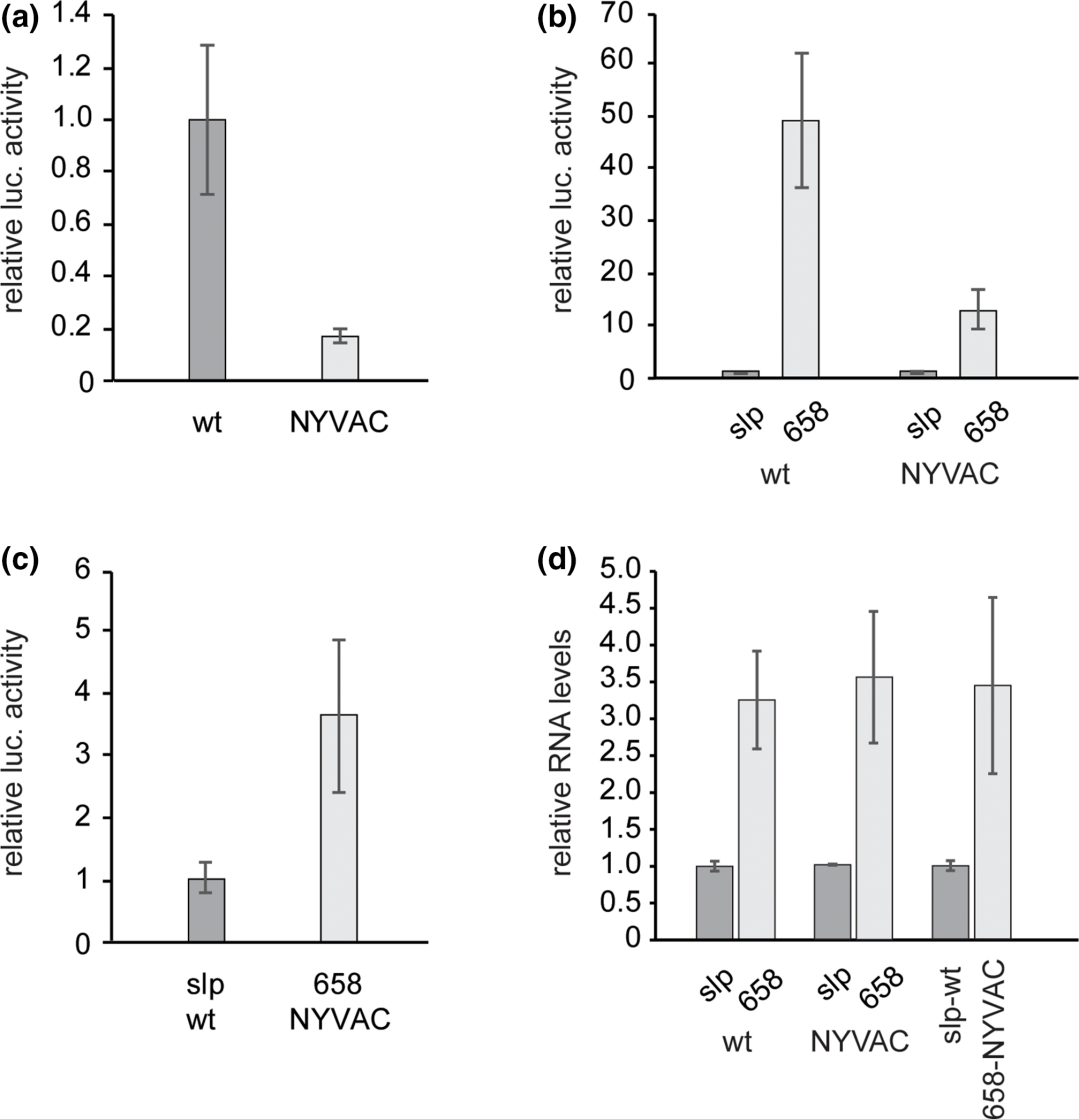
VACV-driven protein expression. (**a**) Luciferase reporter plasmids containing the slp were transfected into HeLa cells which were subsequently infected with either the Copenhagen (wt) or NYVAC strains of VACV. Luciferase protein expression was measured via luciferase assay, and results made relative to those obtained from the wt-infected cells. (**b**) HeLa cells were transfected with luciferase reporter plasmids containing either the slp or hTEE-658 upstream of the coding region. Cells were then infected with either wt or NYVAC virus. Luciferase expression was determined by luciferase assay and results made relative to those obtained from cells containing the plasmid with slp, for the individual viruses. (**c**) Luciferase assay results from cells transfected with either an slp- or hTEE-658-containing plasmid, which were then infected with wt or NYVAC virus, respectively. Expression levels were made relative to those from the slp-plasmid transfected, wt-infected cells. (**d**) mRNA levels for cells transfected with either the slp- or hTEE-658-containing plasmids, and infected with either the wt or NYVAC virus, was determined via quantitative realtime PCR. Levels were made relative to those derived from cells transfected with the slp-containing plasmid in each pairing. All experiments were conducted in triplicate at minimum. Error reported as standard deviation.

Our previous research has demonstrated that hTEE-658 functions to greatly increase protein expression when used in a vaccinia virus (VACV) based cytoplasmic expression system [15]. In this system, the wild-type (wt) Copenhagen strain of VACV was used to facilitate messenger RNA (mRNA) production in the cytoplasm, via the presence of the VACV synthetic late promoter (slp) within the reporter plasmids used. Yet while the use of hTEE-658 has also been evaluated using an attenuated VACV [15], the baseline difference in recombinant protein expression levels between the viral strains had not been quantified in our system. To ascertain the difference in the levels of targeted protein expression between strains of VACV, a direct comparison was performed using a transfect-infect assay with a luciferase reporter plasmid and the Copenhagen and NYVAC viral strains (Fig. 1a). In this assay, the slp was used as the site of transcription initiation and was located immediately upstream of the coding region for the firefly luciferase protein. Based on the resulting luciferase expression, approximately five-fold lower levels of protein are produced when the attenuated NYVAC strain is used in our system (Fig. 1a). This decrease in expression is comparable to previous research with this particular strain of VACV, thus giving confidence in the viability of our system [7].

To quantify the effect of hTEE-658 on protein expression, additional reporter plasmids were generated in which the VACV slp was replaced with the 37-nucleotide functional motif. Therefore, the only promoter site for the VACV RNA polymerase was the hTEE-658 sequence. When used in transfect-infect assays with the wt Copenhagen strain of VACV, a 49-fold increase in luciferase production was observed when hTEE-658 was present, compared to results obtained with the slp-containing plasmid. When the NYVAC strain was used, hTEE-658 driven protein expression increased 12-fold over those of the standard slp (Fig. 1b). Taken together, these results demonstrate that hTEE-658 can increase production of a target protein, when positioned in the leader sequence of the coding region. Furthermore, this increase occurs with both wt and attenuated strains of VACV, although not to the same extent. Control assays confirmed that protein expression does not occur as a result of any nuclear processes in our system, and therefore the observed differences are not the result of cryptic splicing (Fig. S1, available in the online version of this article). The discrepancy in the magnitude of enhancement could be attributed to the overall lower levels of viral replication with NYVAC [2]. An analysis of VACV proteins expressed in both the early and late stages of the viral replication cycle (Fig. S2a, b, respectively), and spanning the incubation time of our transfect-infect assays, confirmed the increased replication capacity of the Copenhagen strain in our system. Taken together with our previously published results indicating a peak in luciferase production around 6 h post-infection [15], this suggests that expression from the plasmids in our system occurs early in the viral replication cycle. Furthermore, as late protein production is not observed with the NYVAC strain at 6 h post-infection (Fig. S2b), yet robust expression of luciferase occurs at this timepoint, it appears that hTEE-658 does not require late VACV protein expression to function. When comparing protein production levels between a standard viral promoter (slp) in combination with wt virus and hTEE-658 with an attenuated VACV (NYVAC), more than a 3.5-fold increase in expression was observed (Fig. 1c). This ability of hTEE-658 to drive protein expression levels with an attenuated virus over those commonly generated with a wt virus system highlights the potential use of this motif to improve vaccines based on attenuated VACV.

As the ability of hTEE-658 has been previously documented to increase both transcription and translation in a VACV system [15], quantitative realtime PCR was used to determine luciferase mRNA levels generated in our transfect-infect assays. Analysis confirmed that the presence of hTEE-658 leads to a three to 3.5-fold increase in mRNA production from a downstream open reading frame, when either the wt or NYVAC virus is used (Fig. 1d). This observation is also consistent when comparing the use of an slp-containing plasmid and the wt virus to the use of an hTEE-658 containing plasmid and NYVAC. This data, taken together with previous observations of increased mRNA levels when hTEE-658 is employed versus the use of other translation enhancing sequences with no demonstrated promoter activity [15], suggest that the increased presence of mRNA within cells is due to the ability of hTEE-658 to drive transcription, as opposed to solely improved mRNA stability due to increased translation along the message. Additionally, while an increase in mRNA production was observed when hTEE-658 was employed, the magnitude of increase was less than that observed when protein production was evaluated (Fig. 1a–c), indicating that the increase in protein expression was not solely due to increased transcriptional activity. This further confirms the capacity of hTEE-658 to increase both transcriptional and translational levels when present in the leader sequence of a coding region, when used in both a wt and attenuated VACV system.

### Leader sequence modifications

**Fig. 2.**
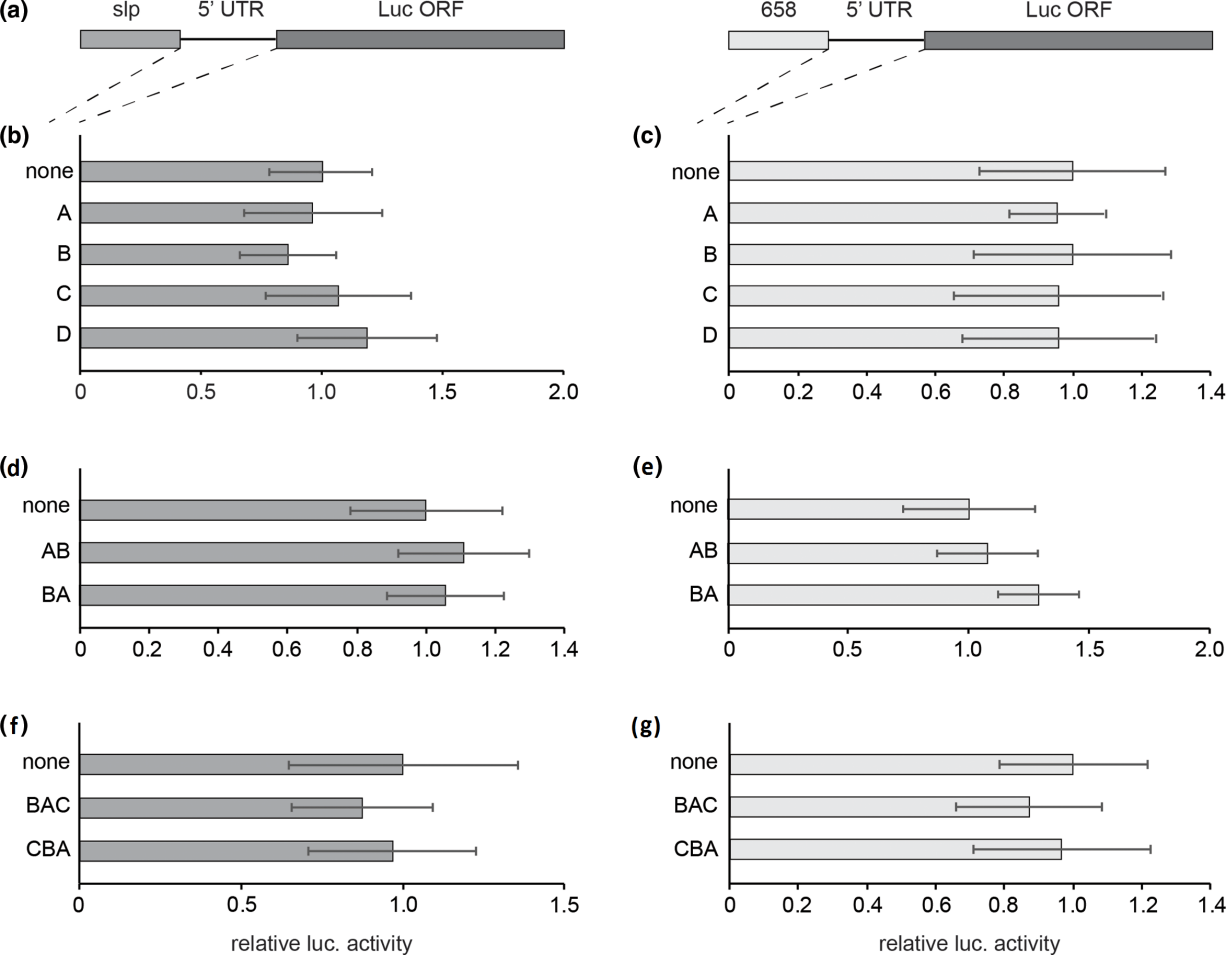
Effect of 5’ leader length on translation levels. (a) Diagram of reporter constructs containing either the slp or hTEE-658 upstream of a leader sequence of varying length, followed by the coding region for the firefly luciferase protein. Luciferase levels were determined by transfect-infect assay using reporters that contain either no inserted leader sequence (none) or a designed sequence consisting of 30 nucleotides (nts) (b, c), 60 nts (d, e) or 90 nts (f, g). Assays were conducted in HeLa cells using the Copenhagen strain of VACV, and in each case results were made relative to those when no leader sequence was present. All experiments were conducted in triplicate at minimum. Error is reported as standard deviation.

In an attempt to improve protein expression driven by hTEE-658, several modifications were made to the 5′ leader sequence. Previous studies have shown that the specific length of the 5′ leader alone is enough to modulate translation. In this research, extending the length of the 5′ UTR resulted in increased protein expression due to additional loading of ribosomes, while shorter sequences led to decreased translation levels [12, 13]. To explore this as a possible mechanism to increase hTEE-658 driven translation, four random sequences were designed to act as spacing in between the TEE and the luciferase coding region within the reporter plasmid, thereby extending the 5′ leader length. These spacer sequences were 30 nucleotides (nt) in length and given the designation A through D (Table S1). When increasing the 5′ leader length, care was taken to avoid the inadvertent creation of upstream start codons (uAUGs), upstream open reading frames (uORF), and secondary structure, as these features can result in poor translation (Table S2) [10, 13]. Given the sole purpose of these spacer sequences was to increase 5′ leader length, a series of control experiments was also conducted to ensure they do not contain any functional activity. In order to ensure the spacer sequences did not contain any promoter activity, they were inserted into a luciferase reporter plasmid that had all known promoter sequences removed. Similar levels of translation were observed when resulting luciferase activity was compared to the results obtained when a promoter-less plasmid with no additional inserted sequence was employed in our system (Fig. S3a). To evaluate translation enhancing capabilities of the spacers, they were inserted in between the slp and the luciferase coding region within our reporter plasmids (Fig. 2a). This placement ensured that the spacer sequences would be present as a 5′ leader on the mRNA generated from the slp.

As a measure for baseline translation activity within our transfect-infect system, a reporter plasmid with the slp immediately upstream of the luciferase coding region was also employed. Similar levels of translation activity were observed when those obtained with the spacers present were compared to baseline levels (Fig. 2b). Taken together, these results indicate that the randomly generated spacer sequences did not facilitate transcriptional or translational activity, thus confirming their role in simply extending the length of the 5′ leader.

To determine the effect of 5′ leader extension on the function of hTEE-658, reporter plasmids containing the motif as a promoter were modified. In these reporters, each of the individual spacers were inserted into an hTEE-658 containing plasmid immediately downstream of the TEE and upstream of the luciferase coding region (Fig. 2a). As a reference, an unmodified hTEE-658 plasmid was also used to highlight the effect of the spacer’s presence. Results of corresponding transfect-infect assays showed no difference in luciferase expression levels with the spacer sequences in place (Fig. 2c). Comparison of results with the promoter-less control plasmids confirms that the activity levels observed when hTEE-658 is upstream of the spacers is not influenced by any promoter activity of the spacers themselves (Fig. S3b). These findings indicate that extension of the leader sequence by 30-nt, with hTEE-658 at the 5′ most end, has neither a positive or negative effect on translation levels.

As previous research has indicated a stepwise increase in 5′ leader length can yield a corresponding upregulation of translation levels [13], we next combined our spacer sequences to form extensions of 60- and 90-nt. The use of repeated spacer sequences was avoided to eliminate the possibility of complications due to homologous recombination involving VACV, which could lead to the excision of duplicated spacers [22]. To generate an extension of 60-nt, spacers A and B were joined in both possible combinations, while an extension of 90-nt was accomplished through the combination of spacers A, B and C, to form BAC and CBA (Table S1). Upon analysis, the new spacer sequence extensions did not lead to the generation of novel translation initiation sites or any significant secondary structure (Table S2). The 60- and 90-nt spacers were inserted into our luciferase reporter plasmids downstream of either the slp or hTEE-658. Analysis of the results from protein expression assays using the slp-containing plasmids indicated very slight differences in translation levels, when compared to results obtained from a plasmid with no 5′ extension (Fig. 2d, f). This suggests that the extended spacers, as with the 30-nt spacers, do not stimulate increased translation levels. When assayed in combination with hTEE-658, only marginal fluctuations in translation activity were observed when the spacers were present, as compared to those when hTEE-658 was assayed alone (Fig. 2e, g). These results indicate that hTEE-658 function is not influenced by its proximity to the downstream coding region, up to a distance of 90-nt from the initiation codon. To confirm that differences in transcriptional levels did not factor into these observations, realtime PCR was performed using mRNA extracted from lysates of cells used in our transfect-infect system. Analysis of luciferase mRNA levels, made relative to those when hTEE-658 was employed alone, displayed only small variations when any of the spacer sequences were present (Fig. S4a). In sum, these results suggest that increasing the distance between hTEE-658 and a downstream coding region does not affect the ability of the motif to drive either VACV-based transcription or translation. Furthermore, simply increasing the length of the 5′ leader sequence did not have the enhancing effect that was observed in other studies [13].

### Translation enhancing element combinations

Previous research has demonstrated that combining known enhancing elements can increase total protein production [23]. To explore this as a possibility to increase hTEE-658 function, several other TEEs from our prior studies were chosen to combine with the motif. These TEEs included hTEE-675,–694, -878,–884 and −985, which were all within the top 10 % of functional sequences identified [14]. Additionally chosen was a highly active 13-nt motif that was originally discovered within a set of TEEs and demonstrated to function independently [18]. Analysis of these functional sequences used in combination with hTEE-658 revealed that none exceeded the 90-nts added by the random spacers nor contained any significant predicted secondary structure. Interestingly, each sequence displayed variations in the number of translation initiation and termination codons, while a few contained complete upstream open reading frames (uORFs) (Table S3). While these features are generally associated with decreased translational efficiency of a downstream open reading frame [10], the selected sequences displayed a drastic ability to increase protein production in previous characterizations [14, 18].

**Fig. 3.**
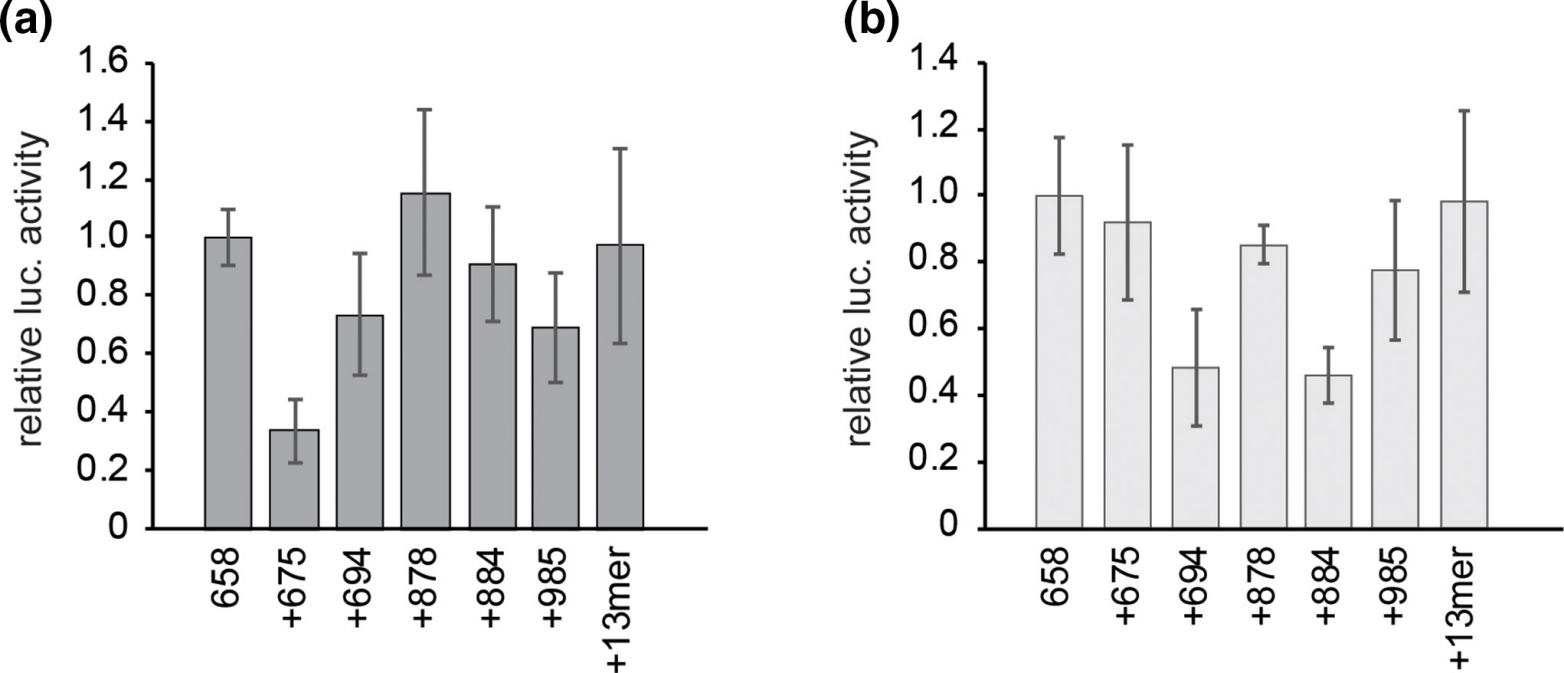
Combinatorial effect of translation enhancing elements. Luciferase production levels when reporter plasmids containing either hTEE-658 alone or in combination with a known translation enhancing element (TEE) were used in a transfect-infect assay with either the Copenhagen (a) or NYVAC (b) strain of VACV. Assays were conducted in HeLa cells, with luciferase assay results made relative to those when only hTEE-658 was present upstream of the luciferase coding region. All experiments were conducted in triplicate at minimum, and error is reported as standard deviation.

The TEEs chosen for combination were inserted into luciferase reporter plasmids immediately downstream of hTEE-658 and immediately upstream of the firefly luciferase coding region. When employed in transfect-infect assays using a wt strain of VACV, the addition of the TEEs did not lead to a significant increase in protein production (Fig. 3a). In fact, the presence of the TEEs led to a decrease in translation levels, with the exception of hTEE-878. In assays using the NYVAC strain of VACV a similar trend was observed, however with slight variations in the effect of the additional TEEs (Fig. 3b). Notably, the presence of hTEE-675 led to approximately three-fold higher levels of protein production in the NYVAC system, compared to the wt system. However, the overall translation levels were no higher than when hTEE-658 alone was present in the reporter plasmid. When present, the 13-nt motif led to the most consistent production of luciferase protein in the different viral systems, with levels of translation comparable to those of hTEE-658 alone in both instances (Fig. 3). To ascertain the effect of the additional TEEs on the ability of hTEE-658 to stimulate VACV-based transcription, quantitative PCR was performed on cell lysates from transfect-infect assays to determine luciferase mRNA levels. In both the wt and NYVAC driven systems, the combination of hTEE-658 with additional sequences yielded only slight alterations to luciferase mRNA production (Fig. S4b). Taken together, the results of combining hTEE-658 with additional translation enhancing sequences did not indicate any increase in transcription or translation of a downstream open reading frame.

### HIV-1 gp120 expression

**Fig. 4.**
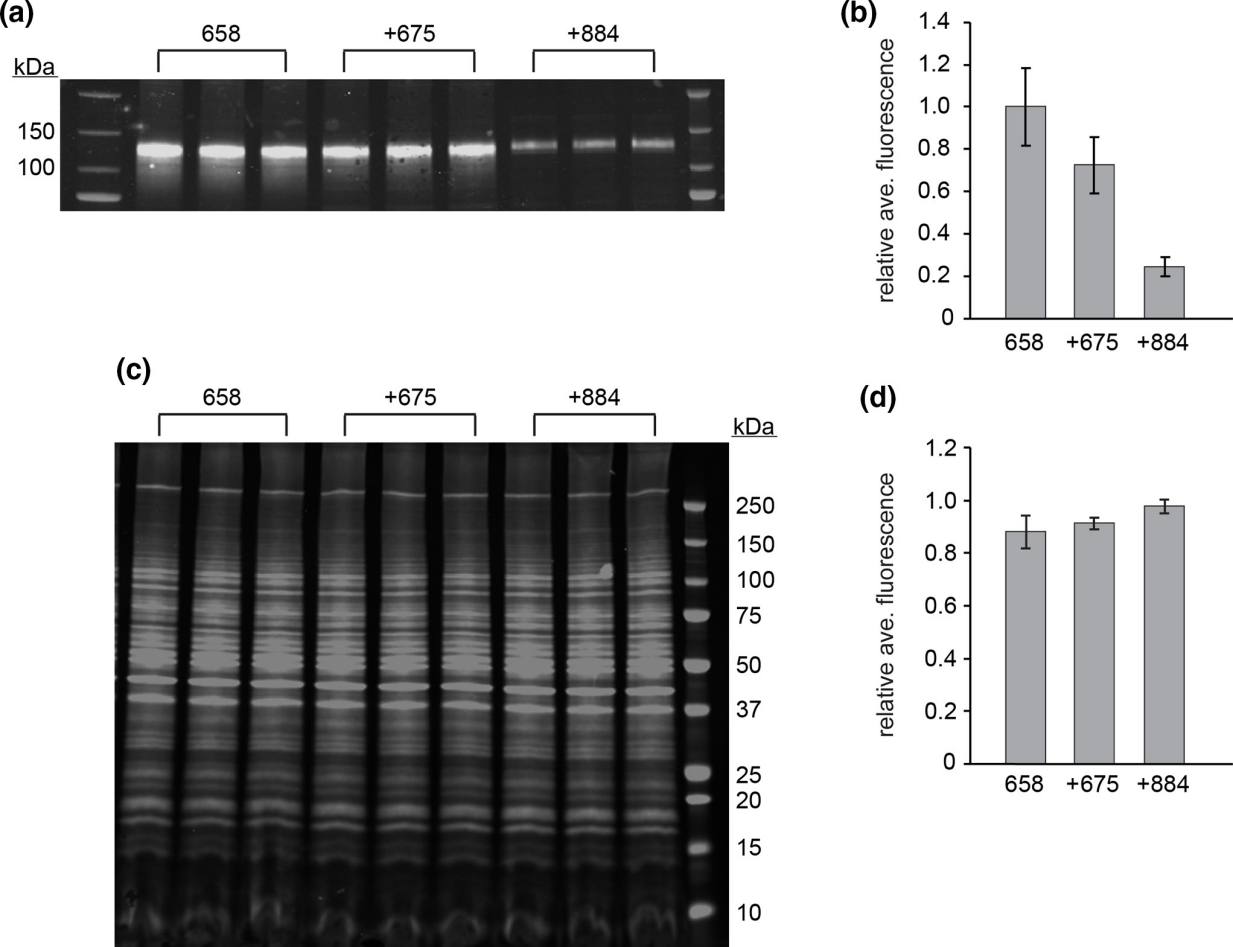
hTEE-658 driven expression of HIV-1 gp120. (**a**) Representative Western blot analysis of lysates from cells transfected with a plasmid containing either hTEE-658 alone or in combination with hTEE-675 or hTEE-884, and infected with the NYVAC strain of VACV. hTEE-658, or the indicated combination, was positioned within the plasmid immediately upstream of the coding region for the gp120 protein of HIV-1. Results of triplicate experiments are shown. (**b**) The normalized fluorescence of each band was determined, and then averaged for each sample. The averages were made relative to those when only hTEE-658 was present within the plasmid. (**c**) Representative Revert total protein staining of samples used for Western blot analysis. (**d**) Fluorescence of each Revert stained lane was determined, and then averaged for each sample. The averages were made relative to the sample with the highest average fluorescence. In all analyses, error is reported as standard deviation.

To evaluate if the results obtained using the luciferase reporter plasmids applied to the expression of other proteins, the luciferase coding region was replaced with the gene for HIV-1 gp120. The plasmids chosen for this analysis included those containing hTEE-658 alone, and those additionally containing hTEE-675 and −884. Evaluation using our NYVAC-based expression system, followed by Western blot analysis, displayed that the gp120 protein was expressed from each plasmid, with the highest intensity bands produced when hTEE-658 was employed alone (Fig. 4a).

For normalization purposes, Revert staining of the Western blot gel was performed (Fig. 4c). This method evaluates the total protein loaded and has been shown to be a more accurate control than traditional detection of a housekeeping protein [24]. Quantification of total protein intensity demonstrated a slight variation in loading, with the largest difference of approximately 15 % less protein loaded for the samples corresponding to when hTEE-658 was used alone (Fig. 4d). Normalization of gp120 expression using the total protein loading data revealed that the combination of another TEE with hTEE-658 reduced protein production (Fig. 4b). These results are consistent with those obtained from the expression of the luciferase protein and suggest that the combination of functional sequences with hTEE-658 may not be a viable method to drive protein levels higher in a VACV-based system. However, gp120 expression driven by hTEE-658 was elevated from 25- to 50-fold higher compared to levels driven by the slp in combination with the wt or NYVAC virus, respectively (Fig. S5). This highlights the potential for the use of hTEE-658 to increase protein production in a NYVAC-based, attenuated VACV vaccine candidate.

### Expression from recombinant virus

**Fig. 5.**
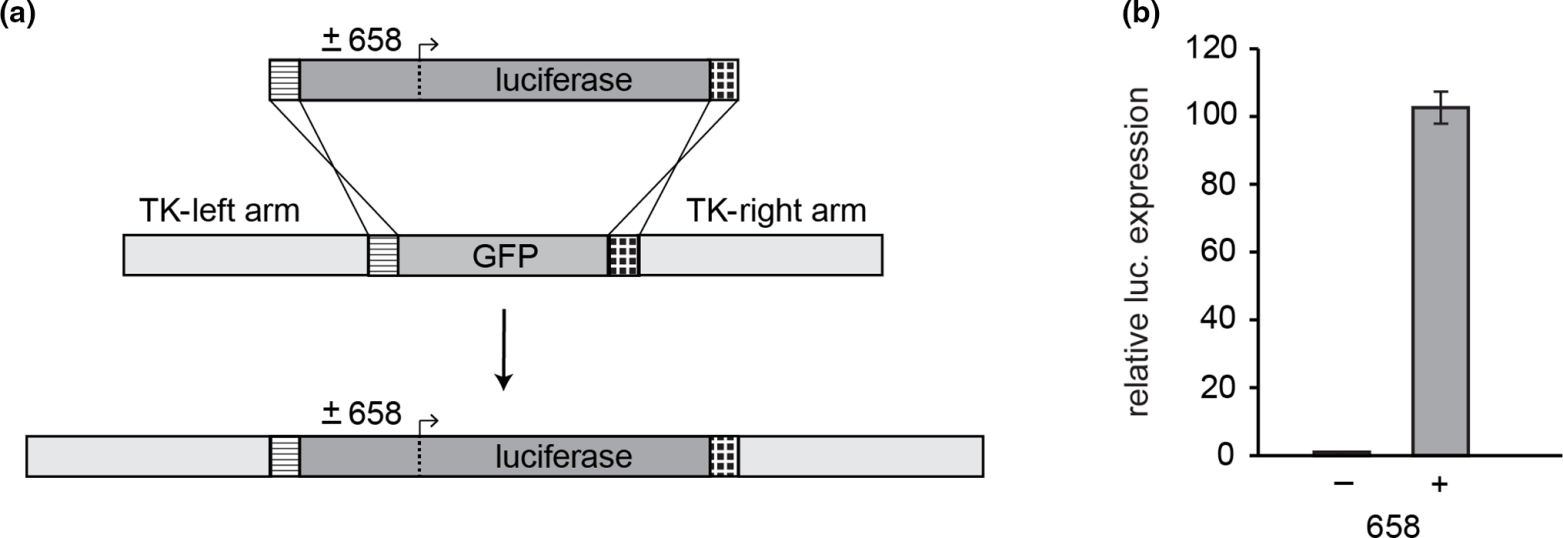
Recombinant virus containing hTEE-658. (**a**) Schematic outlining the use of homologous recombination to insert the firefly luciferase gene, either with or without hTEE-658 immediately upstream, into the TK locus of the NYVAC strain of VACV. In this strategy, the coding region for GFP is replaced with the inserted sequence of interest to aid in the detection of proper insertion. (**b**) Luciferase expression following infection of BSC40 cells with recombinant NYVAC. Luciferase assay results were made relative those when hTEE-658 was not included upstream of the luciferase coding region. Expression experiments were conducted in duplicate, with error reported as standard deviation.

To evaluate whether the increased protein levels observed using hTEE-658 in our transfect-infect expression system would remain consistent when the sequence was incorporated into the genome of an attenuated VACV strain, a recombinant NYVAC virus was constructed. This involved the use of homologous recombination to reciprocally insert the firefly luciferase gene into the thymidine kinase (TK) locus of NYVAC in place of the coding region for green fluorescent protein (GFP), which had been previously inserted for the purposes of viral plaque selection [25]. During the insertion process, hTEE-658 was either included or excluded immediately upstream of the luciferase coding region (Fig. 5a). This resulted in the creation of two recombinant NYVAC strains that expressed firefly luciferase from the viral genome. Analysis of luciferase protein levels following cell culture infection with these viruses indicated that the presence of hTEE-658 increased expression levels approximately 100-fold (Fig. 5b). These observed levels of enhancement were greater than those seen with the transfect-infect system (Fig. 1) and demonstrate that hTEE-658 can serve as a functional element when incorporated into the genome of VACV. Furthermore, these results lend additional support for the use of hTEE-658 to increase expression of a target protein in attenuated VACV vaccine candidates.

## Discussion

Since its use as a vaccine vector in the eradication of smallpox, research on VACV has been targeted towards improving the safety profile of the virus while maintaining immunogenicity. This led to the production of several strains of VACV that were attenuated by varying methods [2]. In general, this attenuation resulted in viral strains that could not replicate as efficiently in mammalian cells and therefore reduced the side effects of exposure [4, 5]. With this success, many attenuated, recombinant vaccine strains of VACV have been developed. These engineered strains have been designed to target a number of different diseases, including those caused by rabies, influenza, human immunodeficiency virus-1, and cancer [26–29]. Yet while a successful VACV-based rabies vaccine has been developed and licensed for use in wildlife [27, 30], attempts to produce a recombinant VACV-based vaccine strain for use in humans has not been accomplished. This is the result of various reasons, depending on the VACV strain used, however most center around the inability to produce an efficient immune response with administration methods that would be amenable to vaccine delivery. Efforts with the VACV strains NYVAC and MVA, two of the most widely studied and promising candidates, have been hindered by the findings that immunization with these attenuated strains requires higher or multiple doses to confer the same protection as replication-competent strains [31, 32]. While methods to combat these drawbacks have largely centered around the modulation of VACV factors that influence the host immune system [8, 9], routes to increase the expression of a targeted recombinant protein from these vaccine candidates remains largely unexplored.

The modulation of protein translation has been achieved by several different strategies in previous research. Studies analyzing 5′ UTR length identified an ideal distance of 77 nucleotides between the 5′ end of the mRNA and the initiation codon, irrespective of the sequence used [13, 33]. The increase in protein production observed at this length was attributed to increased preloading of ribosomal 40S complexes along the span of the UTR. When evaluated in our system however, the length of the leader sequence did not influence translation levels when either a standard VACV promoter or hTEE-658 was positioned at the 5′ end of the RNA. This observation was consistent at every UTR length evaluated, which spanned the ideal distance previously characterized by Kozak. The mechanism behind this discrepancy requires further investigation, however it could be due in part to the involvement of VACV-specific factors and their influence on translation [34, 35]. Other strategies modulating protein production have involved the use of specific sequences to drive the process. One well characterized example of this is the *Gtx* element. This sequence motif, when present in the 5′ UTR of an mRNA, significantly increased protein production, particularly when employed as a concatemer [23]. While this strategy of combining elements was initially appealing, the propensity for VACV to undergo homologous recombination during replication could lead to unwanted modifications that could negatively impact attempts at employing sequentially combined hTEE-658 motifs [22]. To avoid this potential complication, our current approach evaluated combining hTEE-658 with elements previously demonstrated to display translation enhancing capabilities. However, based on our results, this method to increase protein production does not appear to function as previously demonstrated when using hTEE-658 in our system. While the sequences used in combination with hTEE-658 were shown to lack promoter activity in prior studies [14], it is possible that combining these translationally active motifs lead to competition for cellular machinery. There are examples of this documented in the expression of certain cellular genes, which could have hindered any potential increase in protein production in our system [36, 37].

Despite the unsuccessful attempts to increase translation driven by hTEE-658, our current study has demonstrated that this motif is a promising candidate to increase the effectiveness of attenuated VACV-based vaccines. We have shown that when hTEE-658 is positioned upstream of an open reading frame it can lead to protein production in an attenuated virus system that surpasses those of a traditional promoter paired with a wt VACV strain. These findings, therefore, have very positive implications in the use of hTEE-658 to improve recombinant protein production from attenuated VACV-based vaccine candidates. The incorporation of hTEE-658 immediately upstream of the coding region for any desired protein could boost expression of that protein while leaving the attenuated features of the virus unmodified. Based on this study, and in combination with previous research [15], hTEE-658 appears capable of increasing the production of a wide array of target proteins, including those relevant to potential vaccine development. Thus, the attenuation profile of the viral delivery system could be maintained, while increasing the dosage of protein to trigger the host immune response. This increased amount of protein could result in a more robust immune response without the need for an increased dosage of the vaccine candidate itself, as has been observed in previous studies [31]. Furthermore, incorporation of hTEE-658 into the genomic material of a recombinant, attenuated VACV is a straightforward process, as the sequence motif is only 37-nt in length. Using previously published methods as a guide [7, 25], insertion of hTEE-658 into the genome of the attenuated NYVAC strain of VACV was performed with little difficulty. Infections with this recombinant virus, which employed hTEE-658 to drive expression of the firefly luciferase gene, produced substantially elevated levels of protein expression. This demonstrated for the first time that hTEE-658 can function within the genome of VACV to elevate expression of a target protein and underscores its potential use in the development of more effective attenuated VACV vaccine candidates. Additionally, our current results demonstrate this incorporation can occur immediately upstream of the desired open reading frame, thus greatly reducing the amount of genomic sequence added. This is in contrast to other more complicated VACV expression systems which require extensive genomic modification to increase protein production [38]. Our results also indicate some flexibility in the use of hTEE-658, as protein expression levels driven by the motif can remain unchanged when a distance of up to 90 nucleotides is added between the sequence element and the downstream coding region. This depends upon the functionality of the nucleotides added but can yield versatility in the design of expression systems using the motif. Additionally, taken together with our previous research characterizing its use with other strains of VACV [15], our current study suggests using hTEE-658 to increase protein expression should be a viable strategy in combination with a variety of VACV strains. This highlights the versatility of this sequence motif and underscores its broad application in the field of VACV-based vaccine research.

## Supplementary Data

Supplementary material 1

## References

[1] Condit RC, Moussatche N, Traktman P (2006). In a nutshell: structure and assembly of the vaccinia virion. Adv Virus Res.

[2] Jacobs BL, Langland JO, Kibler KV, Denzler KL, White SD (2009). Vaccinia virus vaccines: Past, present and future. Antiviral Res.

[3] Baxby D (1977). The origins of vaccinia virus. J Infect Dis.

[4] Gomez CE, Najera JL, Krupa M, Perdiguero B, Esteban M (2011). MVA and NYVAC as vaccines against emergent infectious diseases and cancer. Curr Gene Ther.

[5] Kenner J, Cameron F, Empig C, Jobes DV, Gurwith M (2006). LC16m8: an attenuated smallpox vaccine. Vaccine.

[6] Tartaglia J, Perkus ME, Taylor J, Norton EK, Audonnet JC (1992). NYVAC: a highly attenuated strain of vaccinia virus. Virology.

[7] Kibler KV, Gomez CE, Perdiguero B, Wong S, Huynh T (2011). Improved NYVAC-based vaccine vectors. PLoS One.

[8] Gómez CE, Perdiguero B, Sánchez-Corzo C, Sorzano COS, Esteban M (2017). Immune Modulation of NYVAC-Based HIV Vaccines by Combined Deletion of Viral Genes that Act on Several Signalling Pathways. Viruses.

[9] Di Pilato M, Mejías-Pérez E, Sorzano COS, Esteban M (2017). Distinct roles of Vaccinia virus NF-κb inhibitor proteins A52, B15, and K7 in the immune response. J Virol.

[10] Babendure JR, Babendure JL, Ding JH, Tsien RY (2006). Control of mammalian translation by mRNA structure near caps. Rna.

[11] Gray NK, Wickens M (1998). Control of translation initiation in animals. Annu Rev Cell Dev Biol.

[12] Kozak M (1991). A short leader sequence impairs the fidelity of initiation by eukaryotic ribosomes. Gene Expr.

[13] Kozak M (1991). Effects of long 5’ leader sequences on initiation by eukaryotic ribosomes *in vitro*. Gene Expr.

[14] Wellensiek BP, Larsen AC, Stephens B, Kukurba K, Waern K (2013). Genome-wide profiling of human cap-independent translation-enhancing elements. Nat Methods.

[15] Wellensiek BP, Larsen AC, Flores J, Jacobs BL, Chaput JC (2013). A leader sequence capable of enhancing RNA expression and protein synthesis in mammalian cells. Protein Sci.

[16] Casey CG, Iskander JK, Roper MH, Mast EE, Wen XJ (2005). Adverse events associated with smallpox vaccination in the United States, January-October 2003. JAMA.

[17] Maduro M (2015). Random DNA sequence generator. http://www.faculty.ucr.edu/~mmaduro/random.htm.

[18] Juba AN, Chaput JC, Wellensiek BP (2018). Exploring the role of AUG triplets in human cap-independent translation enhancing elements. Biochemistry.

[19] Expasy (2015). SIB Swiss Institute of Bioinformatics. https://web.expasy.org/translate.

[20] Markham NR, Zuker M (2008). UNAFold: software for nucleic acid folding and hybridization. Methods Mol Biol.

[21] Jancovich JK, Chapman D, Hansen DT, Robida MD, Loskutov A (2018). Immunization of pigs by DNA Prime and recombinant Vaccinia virus boost to identify and rank African Swine fever virus immunogenic and protective proteins. J Virol.

[22] Ball LA (1987). High-frequency homologous recombination in vaccinia virus DNA. J Virol.

[23] Chappell SA, Edelman GM, Mauro VP (2004). Biochemical and functional analysis of a 9-nt RNA sequence that affects translation efficiency in eukaryotic cells. Proc Natl Acad Sci U S A.

[24] Kirshner ZZ, Gibbs RB (2018). Use of the REVERT total protein stain as a loading control demonstrates significant benefits over the use of housekeeping proteins when analyzing brain homogenates by Western blot. Mol Cell Endocrinol.

[25] Byrd CM, Hruby DE (2004). Construction of recombinant vaccinia virus: cloning into the thymidine kinase locus. Methods Mol Biol.

[26] Gómez CE, Nájera JL, Jiménez V, Wagner R, Graf M (2007). Head-to-head comparison on the immunogenicity of two HIV/AIDS vaccine candidates based on the attenuated poxvirus strains MVA and NYVAC co-expressing in a single locus the HIV-1BX08 gp120 and HIV-1(IIIB) Gag-Pol-Nef proteins of clade B. Vaccine.

[27] Esposito J, Brechling K, Baer G, Moss B (1987). Vaccinia virus recombinants expressing rabiesvirus glycoprotein protect against rabies. Virus Genes.

[28] Wang W, Huang B, Wang X, Tan W, Ruan L (2019). Improving cross-protection against influenza virus using recombinant Vaccinia vaccine expressing NP and M2 ectodomain tandem repeats. Virol Sin.

[29] Haddad D (2017). Genetically engineered Vaccinia viruses as agents for cancer treatment, imaging, and transgene delivery. Front Oncol.

[30] Maki J, Guiot AL, Aubert M, Brochier B, Cliquet F (2017). Oral vaccination of wildlife using a vaccinia-rabies-glycoprotein recombinant virus vaccine (RABORAL V-RG®): a global review. Vet Res.

[31] Sumner RP, Ren H, Ferguson BJ, Smith GL (2016). Increased attenuation but decreased immunogenicity by deletion of multiple vaccinia virus immunomodulators. Vaccine.

[32] Belyakov IM, Earl P, Dzutsev A, Kuznetsov VA, Lemon M (2003). Shared modes of protection against poxvirus infection by attenuated and conventional smallpox vaccine viruses. Proc Natl Acad Sci U S A.

[33] Kozak M (1988). Leader length and secondary structure modulate mRNA function under conditions of stress. Mol Cell Biol.

[34] Bablanian R, Goswami SK, Esteban M, Banerjee AK, Merrick WC (1991). Mechanism of selective translation of vaccinia virus mRNAs: differential role of poly(A) and initiation factors in the translation of viral and cellular mRNAs. J Virol.

[35] Strnadova P, Ren H, Valentine R, Mazzon M, Sweeney TR (2015). Inhibition of translation initiation by protein 169: A Vaccinia virus strategy to suppress innate and adaptive immunity and alter virus virulence. PLoS Pathog.

[36] Chu D, Barnes DJ, von der Haar T (2011). The role of tRNA and ribosome competition in coupling the expression of different mRNAs in *Saccharomyces cerevisiae*. Nucleic Acids Res.

[37] De Vos D, Bruggeman FJ, Westerhoff HV, Bakker BM (2011). How molecular competition influences fluxes in gene expression networks. PLoS One.

[38] Hebben M, Brants J, Birck C, Samama JP, Wasylyk B (2007). High level protein expression in mammalian cells using a safe viral vector: Modified vaccinia virus Ankara. Protein Expr Purif.

