# Supplementary material for: Optimization of translation enhancing element use to increase protein expression in a vaccinia virus system
Source: J Gen Virol. 2021 Aug 12;102(8):001624. doi: 10.1099/jgv.0.001624 (PMC8513643; doi:10.1099/jgv.0.001624)
Supplement: Supplementary material 1 [file jgv-102-1624-s001.pdf]

Supplementary Information For

Optimization of Translation Enhancing Element use to Increase Protein Expression in a Vaccinia Virus System

Harold B. Richard Jr.[1,*], Stephanie Minder[1,*], Amandeep Sidhu[2], Amber N. Juba[1], James K. Jancovich[3], Bertram L. Jacobs[4,5] and Brian P. Wellensiek[1,#]

[1]Biomedical Sciences Program, College of Graduate Studies, [2]Arizona College of Osteopathic Medicine, Midwestern University, Glendale, AZ 85308, USA. [3]Department of Biological Sciences, California State University San Marcos, San Marcos, CA 92078. [4]Biodesign Center for Immunotherapy, Vaccines and Virotherapy, [5]School of Life Sciences, Arizona State University, Tempe, AZ 85287, USA.

[*]Authors contributed equally to this work

Corresponding author: [#]bwelle@midwestern.edu

Includes:

Supplementary figures 1 through 5

Supplementary tables 1 through 3

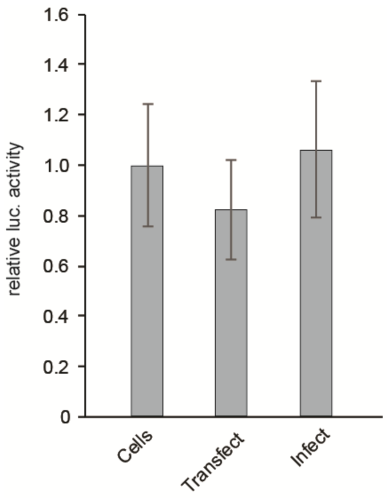

	Relative ratio
658	5607 ± 806
cells	1.0 ± 0.24

Supplementary Figure 1. Transfect-infect assay nuclear expression controls. Luciferase expression was determined via luciferase assay on lysates from HeLa cells alone (cells), from HeLa cells only transfected with a luciferase reporter plasmid containing hTEE-658 (transfect) or from those that had been infected with VACV (infect). The inset table displays luciferase assay results from transfect-infect assays using a plasmid containing hTEE-658 upstream of the luciferase protein coding region, made relative to results when HeLa cells alone were assayed. All assays were done in triplicate, with error reported as standard deviation.

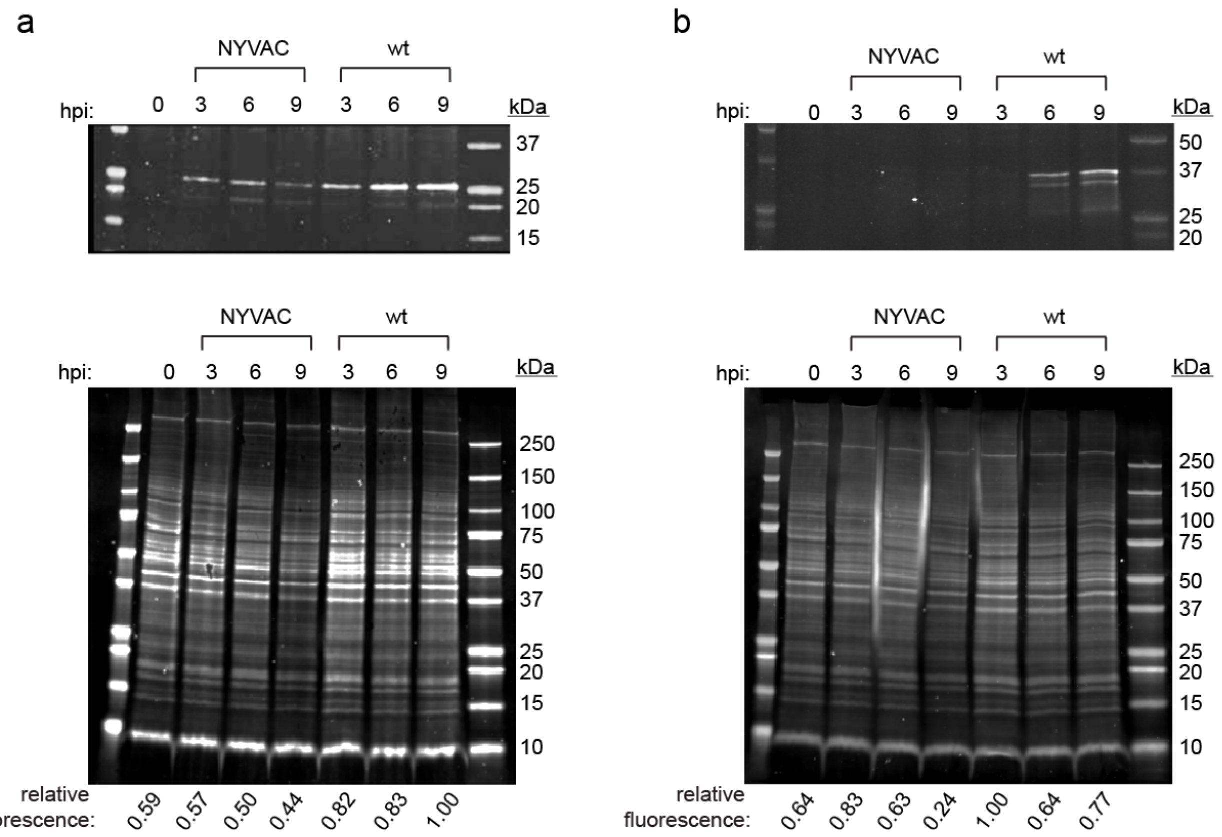

Supplementary Figure 2. Time course of VACV protein expression. Representative western blots analyzing the expression of early (a) and late (b) VACV proteins within HeLa cell lysates. Cells were infected with either the NYVAC or Copenhagen (wt) strain of VACV, and lysates were generated at the indicated hours post-infection (hpi). Specific antibodies were used to detect viral proteins (upper panels), while the Revert™ total protein stain was used as a loading control to determine total protein per well (lower panels). For comparison, total protein levels were made relative to those corresponding to the lane with the highest fluorescence, and the relative fluorescence of each lane indicated below the image.

Supplementary Table 1. Sequences of randomly generated spacers.

**Spacer**	**Sequence**
A	5’ TTACAGAAAAAATTGGAAAAATACGAGCCA 3’
B	5’ ATATCCAGGCTACATACATACCTCTCCTAT 3’
C	5’ GGTATTGTATATTGGAGAATTTGTACTATA 3’
D	5’ TACCTATACACAGTGCAAACTATACCAAAA 3’
AB	5’ TTACAGAAAAAATTGGAAAAATACGAGCCAATATCCAGGCTACAT ACATACCTCTCCTAT 3’
BA	5’ ATATCCAGGCTACATACATACCTCTCCTATTTACAGAAAAAATTGGA AAAATACGAGCCA 3’
BAC	5’ ATATCCAGGCTACATACATACCTCTCCTATTTACAGAAAAAATTGGAAA AATACGAGCCAGGTATTGTATATTGGAGAATTTGTACTATA 3’
CBA	5’ GGTATTGTATATTGGAGAATTTGTACTATAATATCCAGGCTACATACATA CCTCTCCTATTTACAGAAAAAATTGGAAAAATACGAGCCA 3’

Supplementary Table 2. Analysis of randomly generated spacer sequences.

**Spacer**	**GC content**	**#ATGs**	**ΔG**	**ΔG + 658***
A	30%	0	0.86	-0.29
B	40%	0	0.74	0.59
C	26.7%	0	1.29	0.7
D	33.3%	0	-1.25	-1.25
AB	35%	0	-1.74	-2.03
BA	35%	0	0.43	0.43
BAC	32.2%	0	0.09	0.09
CBA	32.2%	0	0.43	0.43

* free energy determined when hTEE-658 is immediately upstream of the spacer

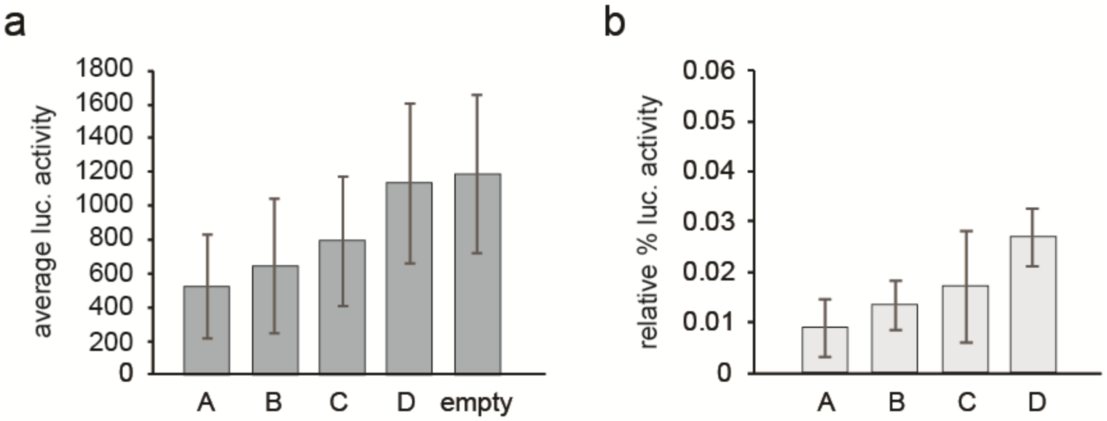

Supplementary Figure 3. Promoter control for randomly generated spacer sequences. The randomly generated spacer sequences were inserted into plasmids which had all known promoter sequences removed. (a) Luciferase activity measured following the use of the promoter-less plasmids in transfect-infect assays using the Copenhagen strain of VACV. Results were compared to those when no additional sequences were added to the plasmids following promoter removal (empty). (b) Luciferase assay results obtained when those generated using the promoter-less plasmids were made relative to those when the corresponding plasmid containing hTEE-658 upstream of the spacer were used. All assays were conducted in triplicate at a minimum, with error reported as standard deviation.

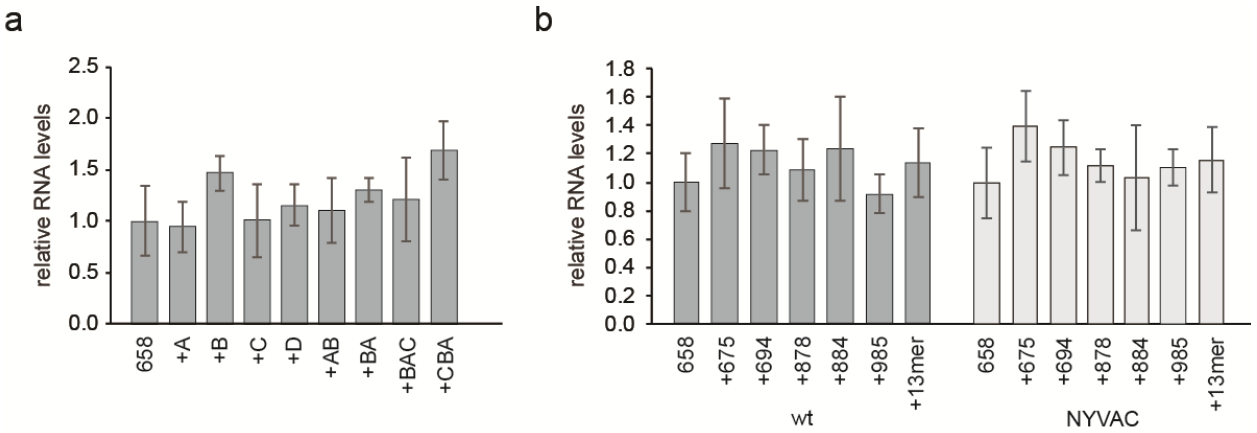

Supplementary Figure 4. Transcription levels. Normalized luciferase mRNA levels within HeLa cells when plasmids containing hTEE-658 and either the indicated random spacers and the Copenhagen strain of VACV (a) or the indicated various TEEs and the Copenhagen (wt) or NYVAC strains of VACV (b) were used in transfect-infect assays. RNA levels were determined via realtime PCR, and results were made relative to those when only hTEE-658 was present in the reporter plasmid. All assays were conducted in triplicate, with error reported as standard deviation.

Supplementary Table 3. Analysis of TEEs used in combination with hTEE-658.

**TEE**	**Length***	**GC content**	**#ATGs**	**#stop^**	**#uORF†**	**ΔG**	**ΔG + 658‡**
675	78	39.7%	0	3	0	0.18	-0.21
694	75	38.6%	1	2	0	-0.82	-1.2
878	90	36.6%	5	4	0	-1.8	-2.19
884	87	33.3%	6	4	1	-0.76	-1.15
985	84	29.7%	3	5	4	-0.25	-0.64
13mer	13	15.4%	1	1	0	1.08	-0.39

* overall sequence length in nucleotides
^ the total number of stop codons combined (TAA, TAG, TGA)
† the total number of complete open reading frames found within the sequence
‡ free energy determined when hTEE-658 is immediately upstream of the TEE

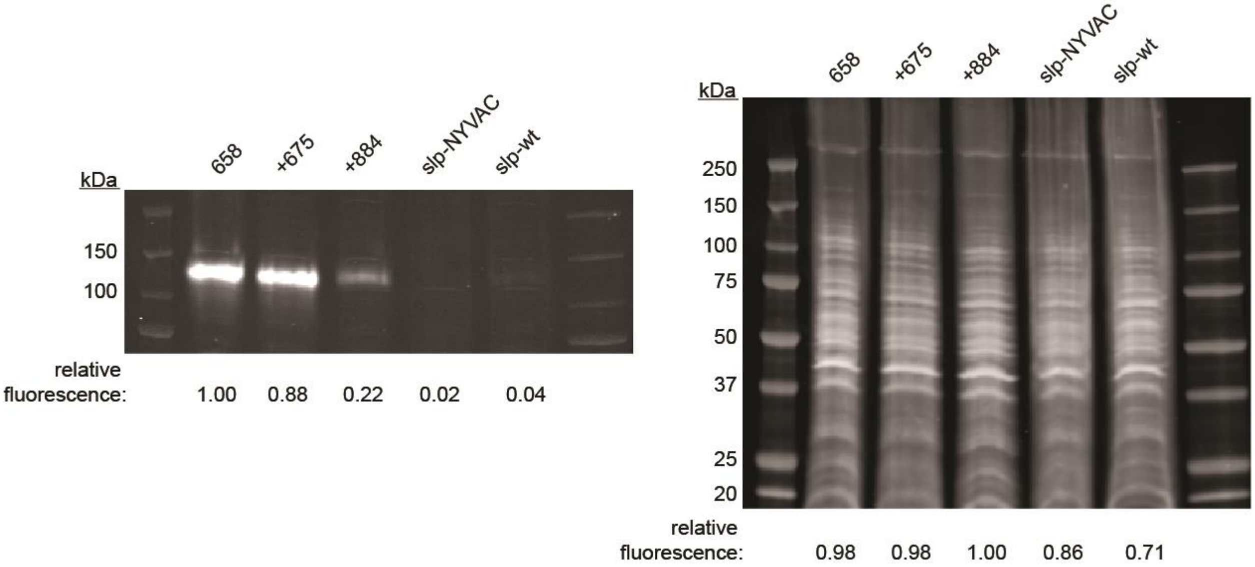

Supplementary Figure 5. 658 enhancement of gp120 expression. (left) Western blot analysis of lysates from cells transfected with a plasmid containing either hTEE-658 alone or in combination with hTEE-675 or hTEE-884 and infected with the NYVAC strain of VACV. Lysates from cells transfected with a plasmid containing the slp alone and infected with either the wt or NYVAC strain of VACV were analyzed for comparison. The relative fluorescence of the gp120 band for each sample was determined and normalized to Revert™ total protein fluorescence of the corresponding well (right).
